# Improved Machine Learning-Based Predictive Models for Breast Cancer Diagnosis

**DOI:** 10.3390/ijerph19063211

**Published:** 2022-03-09

**Authors:** Abdur Rasool, Chayut Bunterngchit, Luo Tiejian, Md. Ruhul Islam, Qiang Qu, Qingshan Jiang

**Affiliations:** 1 University of Chinese Academy of Sciences, Beijing 101408, China; rasool@siat.ac.cn (A.R.); chayutb@ia.ac.cn (C.B.); 2 Shenzhen Key Lab for High Performance Data Mining, Shenzhen Institute of Advanced Technology, Chinese Academy of Sciences, Shenzhen 518055, China; qiang@siat.ac.cn; 3 State Key Laboratory of Management and Control for Complex Systems, Institute of Automation, Chinese Academy of Sciences, Beijing 100190, China; 4 Department of Electrical Engineering and Computer Science, University of Stavanger, 4044 Stavanger, Norway; mr.islam@stud.uis.no

**Keywords:** machine learning models, data exploratory techniques, breast cancer diagnosis, tumors classification

## Abstract

Breast cancer death rates are higher than any other cancer in American women. Machine learning-based predictive models promise earlier detection techniques for breast cancer diagnosis. However, making an evaluation for models that efficiently diagnose cancer is still challenging. In this work, we proposed data exploratory techniques (DET) and developed four different predictive models to improve breast cancer diagnostic accuracy. Prior to models, four-layered essential DET, e.g., feature distribution, correlation, elimination, and hyperparameter optimization, were deep-dived to identify the robust feature classification into malignant and benign classes. These proposed techniques and classifiers were implemented on the Wisconsin Diagnostic Breast Cancer (WDBC) and Breast Cancer Coimbra Dataset (BCCD) datasets. Standard performance metrics, including confusion matrices and K-fold cross-validation techniques, were applied to assess each classifier’s efficiency and training time. The models’ diagnostic capability improved with our DET, i.e., polynomial SVM gained 99.3%, LR with 98.06%, KNN acquired 97.35%, and EC achieved 97.61% accuracy with the WDBC dataset. We also compared our significant results with previous studies in terms of accuracy. The implementation procedure and findings can guide physicians to adopt an effective model for a practical understanding and prognosis of breast cancer tumors.

## 1. Introduction

Breast cancer (BC) is the world’s leading cause of death in women after lung cancer, with approximately 2,261,419 new cases and 684,996 new deaths in 2020 [1]. In the United States, 281,550 new cases were diagnosed with breast cancer, and 43,600 deaths were reported in the females during 2021 [2]. Breast cancer is a type of cancer that originates from breast tissue, most generally from the internal layer of the milk conduit or the lobules that provide milk to the milk conduit. Cancer cells arise from natural cells due to modification or mutation of deoxyribonucleic acid (DNA) and ribonucleic acid (RNA). These modifications or mutations may occur spontaneously as a result of the increase in entropy, or they may be triggered by other factors. For example, electromagnetic radiation (X-rays, microwaves, ultraviolet-rays, gamma-rays, et cetera), nuclear radiation, bacteria, viruses, fungi, parasites, chemicals in the air, heat, food, water, free radicals, mechanical cell-level injury, evolution, and aging of DNA and RNA [3]. In general, benign and malignant are two classes of tumors. Although benign is not life-threatening and cancerous, it may boost the chances of breast cancer risk. In contrast, malignant is more alarming and cancerous tumors. A study performed breast cancer detection and reported 20% of women died due to malignant tumors [4].

These studies emphasize the diagnosis of tumors, and recently, it is a trending biomedical issue. The researchers are employing data mining (DM) and machine learning (ML) technologies for breast cancer prediction [5]. Classifier-based prediction models on DM and ML can limit the diagnosis errors and enhance the efficiency of a cancer diagnosis. DM is an extensive combination of different approaches to discover hidden knowledge and information from large-scale datasets that are difficult to analyze directly. It has been broadly used in the implementation of the prediction system for various diseases, such as heart disease [6], lung cancer [7], and thyroid cancer [8]. DM and ML techniques have been embedded for diagnosing breast cancer with computer-aided systems [9], and fuzzy-genetics [10]. The results of these studies successfully classify the features into two types of tumors by the evaluation of classifier and predicting the incoming tumor based on previous data.

In the literature, a research study proved that breast cancer prediction with machine learning classifiers in the early phases does not just increase the survival chances but can control the diffusion of cancerous cells in the body [11]. For instance, a study used the support vector machine (SVM) based method for breast cancer diagnosis and achieved practical results in prediction [12]. Similarly, Furey et al. [13] also employed SVM for cancer tissue classification with a linear kernel and attained a 93.4% accuracy. Later, this work was extended by Zheng et al. (2014) by delivering a K-SVM hybrid model for Wisconsin Diagnostic Breast Cancer (WDBC) dataset classification and acquiring 97% accuracy [14]. Meanwhile, some other researchers worked on different classifiers, such as Seddik et al. (2015), who proposed a method based on tumor variables for a binary logistic model to diagnose breast cancer WDBC data and secure good results [15]. Likewise, Mert et al. used a k-nearest neighbor (KNN) classifier to predict breast cancer by designing a feature reduction method with independent component analysis. It distributed the features with reduced one feature (1C) and 30 features and computed the performance, and attained 91% accuracy [16].

Apart from these advantageous accuracies with different classifiers and methods, these studies mentioned above have not considered the data exploratory techniques, which enable the data mining techniques to be more robust to acquire efficient performance. Due to the absence of such essential techniques, various studies [16,17,18,19] face the accuracy limitation of ML classifiers. Meanwhile, the confusion matrices misdiagnosed the malignant and benign classes in those studies due to the incorrect prediction of true negative and false negative matrices. Another defect was found in those previous studies that used criteria to assess the feature training with nonlinear classification. However, the performance of model execution time increases rapidly with the number of features [20]. As a result, the prediction model becomes slower, affecting the diagnosis accuracy. In contrast, the model’s accuracy and time complexity are critical issues for the data analyst and physician. These problems, as mentioned above, and findings motivated us to pursue a new study for breast cancer diagnosis by proposing data mining techniques with different machine learning models.

In this research, four different prediction models were formulated with four machine learning algorithms (SVM, KNN, logistic regression (LR), and ensemble classifier (EC)) to deal with a massive volume of tumor features for the extraction of essential information for the diagnosis of breast cancer. The objective was to explore an accurate and efficient prediction model for tumor classification by using data mining techniques. It proposes four-layered significant data exploratory techniques (DET), including feature distribution, elimination, and constructing a hyperparameter for the practical analysis of Wisconsin Diagnostic Breast Cancer (WDBC) and Breast Cancer Coimbra Dataset (BCCD). These techniques enabled the machine learning predictive models to improve accuracy and enhance diagnostic efficiency. In the absence of these techniques, we observed some literature suffers from accuracy limitations. Although image data are more reasonable for breast cancer detection, we have not considered them in this work due to the targeted WDBC and BCCD datasets to apply the intelligent ML classifiers. It presents a framework by integrating DET and predictive models to explore the implementation method for breast cancer diagnosis. The tumor features can be presented in many details, which produces redundant information. Such features lead to tedious outcomes due to high computation times. As a result, our fundamental goal was not only to investigate the effective predictive model with attainable accuracy but also one with time complexity for the cancer diagnosis. The deliberation of time efficiency will enable our models to extract and mine vital information from a vast dataset by finding correlations and eliminating the features. The results presented satisfactory accuracy for the breast cancer diagnosis with the lowest computation time, which signifies the quality of our study as compared to others. This work will enable a data analyst to apply an intelligent machine learning model to analyze breast cancer data. Likewise, a physician would diagnose breast cancer precisely by the tumor classification. As the dataset is available publicly, we uploaded our code on GitHub (https://github.com/abdul-rasool/Improved-machine-learning-based-Predictive-Models-for-Breast-Cancer-Diagnosis (accessed on 11 November 2021)) to assist data analysts and physicians in further advancement and apply it in real-time. As summarized, the following are the significant contributions of this study:

- We investigated four prediction models (SVM, LR, KNN, and EC) with the WDBC and BCCD breast cancer datasets, which reached the next level of quality by diagnosing the tumor and classifying it into benign and malignant.
- It proposes four-layered data exploratory techniques before implementing four ML classifiers as prediction models. These techniques enable the predictive models to acquire peak accuracies for breast cancer diagnosis.
- We set up experiments to validate the models’ prediction and classification accuracy with regard to time complexity and deliver comparative analysis with state-of-the-art studies and various evaluation matrices.

The rest of the article is organized as follows: Section 2 expands on the literature reviews; Section 3 explains the preliminary part for the introduction of proposed prediction models; Section 4 introduces the proposed methodology; Section 5 deals with the evaluation of the results; Section 6 deliberates the discussion, and Section 7 provides the conclusion.

## 2. Related Work

Breast cancer disease causes a massive number of deaths in the world. After the traditional cancer detection methods, the latest technologies enable experts with numerous adaptive methods to discover breast cancer in women. Along with the new technologies, various data science (DS) techniques assist in cancer-based data collection and evaluation to predict this deadly disease. Machine learning algorithms have been successfully applied to cancer-based data analysis among these DS technologies. For example, research [21] was conducted to prove that these machine learning algorithms can improve diagnostic accuracy. It turns out that a 79.97% diagnostic accuracy was achieved by an expert physician. However, 91.1% correct predictions were attained with machine learning.

In the last couple of decades, machine learning applications in the medical field have gradually increased. However, the data collected from the patients and evaluation by the medical expert are the essential factors for diagnosis. The machine learning classifiers have aided in minimizing human errors and delivered prompt analysis of medical data with greater depth [22]. There are several machine learning classifiers for data modeling and prediction; in our work, we employed support vector machine (SVM), logistic regression (LR), k-nearest neighbor (KNN), and ensemble classifier (EC) for breast cancer prediction.

In previous studies, SVM was a widely implemented machine learning algorithm in the diagnosis domain of breast cancer due to its highest prediction accuracy. For instance, Furey et al. (2000) presented SVM with a linear kernel for cancer tissue diagnosis and reached acceptable accuracy [13]. Similarly, Polat et al. (2007) used the least square SVM for breast cancer prediction to eliminate redundant features and secured a 98.53% accuracy. It was suggested that least square SVM assisted in model training with linear equations [23]. However, his method did not deliver the feature selection process. The author [24] delivered a distributed database for multi-active features to integrate different technologies. In 2010, Prasad and Jain et al. [25] proposed a heuristic model for feature subset to train the SVM classifier. It classifies the breast cancer data into two different classes with 91.7% accuracy. However, this accuracy can be adequately improved if the author employs the feature eradication method to get rid of the noise data.

Similarly, Zheng et al. (2014) proposed a hybrid model combining K-mean and SVM classifiers. This model objective was to diagnose the tumor features from the Wisconsin Diagnostic Breast Cancer (WDBC) dataset by employing the feature selection and extraction method. A K-mean classifier was employed to identify the benign and malignant tumor patterns. The generated patterns are computed and considered as new patterns for the training of the SVM model. Then, SVM is executed for the prediction of incoming tumors. The employment of their hybrid model improved the accuracy to 97%. However, the data exploratory techniques are the fundamental tasks for the data preparation, which have not been adequately addressed to train the proposed model [14].

Apart from the SVM, Lim and Sohn et al. (2013) performed logistic regression (LR) with optimal parameters on the Wisconsin Original Breast Cancer (WOBC) and WDBC datasets. It achieved 97.8% sufficient accuracy for the WOBC dataset and 93.8% accuracy for the WDBC dataset with optimized feature sets [26]. Similarly, Seddik et al. (2015) presented a binary logistic model for the diagnosis of breast cancer data based on variables with tumor image characteristics. The proposed model classifies the WDBC data into malignant and benign and accomplished the 98% average classification accuracy. This regression model found that area, texture, concavity, and symmetry are significant WDBC features [15].

Previous literature reviews found numerous studies based on the SVM model for breast cancer detection; however, few were based on others. For example, A. Mert et al. (2015) delivered a feature reduction method with independent component analysis to predict breast cancer. It utilized the k-nearest neighbor (KNN) classifier to categorize the WDBC features efficiently with a reduced one feature (1C) and 30 features. It computed the performance with different matrices and attained 91% accuracy [16]. Later, this study was further improved by Rajaguru et al. (2019), who tackled the breast cancer prediction challenge by implementing the KNN and decision tree (DT) machine learning algorithms to classify the WDBC features. It used a traditional principal component analysis (PCA) feature selection method for the feature categorization and found that KNN outperformed the DT [18]. In another study conducted by Yang and Xu et al. (2019), KNN achieved 96.4% accuracy with the same feature selection method (PCA) [27]. Recently, work has involved considering KNN efficiency by the k values and many distance functions of KNN to find its effectiveness with two different breast cancer datasets. It involves the three different types of the experiment: KNN without feature selection, with linear SVM, and with Chi-square-based features. It indicated that the third technique, Chi-square-based feature selection, succeeded in accomplishing the highest accuracy on both datasets with Manhattan or Canberra distance functions [19].

As for the fourth prediction model, named ensemble classifier (EC) with the voting technique, few studies consider this approach for breast cancer prediction. For instance, M. Abdar et al. (2020) proposed an ensemble method by vote/voting classifier to detect benign tumors from malignant breast cancer. It established a two-layer voting classifier for two or three different machine learning algorithms. The results of these voting techniques disclosed the adequate performance of the simple classification algorithm [5]. From these studies, we got the motivation to conduct experiments based on voting classifiers with different machine learning techniques. However, none of the above approaches has utilized the feature correlation and elimination for the given breast cancer dataset to the best of our knowledge. These studies conducted experiments to classify the cancer features, which is still a challenging issue. Recently, in Nature Cancer, a study presented an approach to classify cancer into normal and tumor tissues [28]. Meanwhile, many studies have utilized the SVM classifier for breast cancer prediction, while a few of them used only one classifier in experiments. However, there is still a demand to explore the efficient classifier for breast cancer prediction with more effective methods [5,14,15,18]. This study performed four different prediction models with sufficient data mining exploratory techniques to diagnose breast cancer.

## 3. Preliminary

This section deliberates data information and evaluation matrices for this study.

### 3.1. Data Description

In this research, the experiments were performed on two different datasets: WDBC and BCCD. The selection reason for these datasets is it is extensively used in numerous studies [16,28,29,30]. Moreover, those ML models that deliver adequate accuracy with the binary dataset were trained. The detailed introduction and particular selection reason of these datasets are given below:

**Wisconsin Diagnostic Breast Cancer (WDBC):** The WDBC dataset consists of 10 features of breast tumor, and the result in the data were taken from 569 patients. Dr. William H. Wolberg distributed it at the General Surgery Department, University of Wisconsin-Madison, USA. It can be obtained via the file transfer protocol (FTP) from this link (https://ftp.cs.wisc.edu/math-prog/cpo-dataset/machine-learn/cancer/WDBC/ (accessed on 11 November 2021)). This dataset was created using fluid samples taken from patients’ solid breast masses. Then, software called Xcyt was used to perform cytological feature analysis based on the digital scan. This software applies a curve-fitting algorithm to calculate ten features by returning each feature’s mean value, worst value, and standard error (SE) value. Thus, there were 30 values in total for each sample, to which we have added an ID column to differentiate these samples. Finally, the diagnosis result of each sample, which consisted of malignant (M) and benign (B), was also added. In conclusion, the dataset contained 32 attributes (ID, diagnosis, and 30 input features) and 569 instances. Features of each sample were radius (mean of distances from the center to points on the perimeter), texture (standard deviation of gray-scale values), perimeter, area, smoothness (local variation in radius lengths), compactness (calculated by, $\frac{perimeter^{2}}{area-1}$ concavity (severity of concave portions of the contour), concave points (number of concave portions of the contour), symmetry, and fractal dimension (calculated by coastline approximation −1).

The first column of the dataset, ID, was not considered and was dropped from the analysis. The second column, which is the diagnosis, will become the target of the study. The third to the thirty-second column contains the mean, SE, and worst values of each feature, shown in Table 1. For instance, feature number 2 is Texture means; feature number 12 is Texture SE; and feature number 22 is Texture worst.

**Breast Cancer Coimbra Dataset (BCCD):** This dataset consists of nine predictors and a binary dependent variable indicating the presence or absence of breast cancer. It can be downloaded from this link (https://archive.ics.uci.edu/ml/datasets/Breast+Cancer+Coimbra (accessed on 11 November 2021)). The predictors are simple parameters that can be collected from routine blood analysis. The nine predictors are Age (years), BMI (kg/m^2^), Glucose (mg/dL), Insulin (μU/mL), Homeostasis Model Assessment (HOMA), Serum value of Leptin (ng/mL), Adiponectin (μg/mL), Resistin (ng/mL), and Chemokine Monocyte Chemoattractant Protein 1 (MCP-1) (pg/dL). The dataset was gathered by the Gynecology Department of the University Hospital Center of Coimbra in Portugal between 2009 and 2013. It was collected from naïve data (the data were collected before the treatment) of 64 women diagnosed with breast cancer and 52 healthy women (a total of 116 instances).

### 3.2. Performance Evaluations Matrices

In this research, we compared four cross-validation matrices: precision, recall, F1 score, and accuracy. These matrices can be calculated by using the values in the confusion matrix, which are true positive (TP)—the prediction is yes, and the actual data is also yes; true negative (TN)—the prediction is no, and the actual data is also no; false positive (FP)—the prediction is yes, but the actual data is no; and false negative (FN)—the prediction is no, but the actual data is yes. Precision, recall, F1 score, and accuracy can be calculated as in the equations below [20]:

(1) $$precision\left(P\right)=\frac{TP}{Tp+FP}$$

(2) $$Recall\left(R\right)=\frac{TP}{Tp+FN}$$

(3) $$F1score=\frac{2\times P\times R}{P+R}$$

(4) $$Accuracy\left(A\right)=\frac{TP+TN}{TP+TN+FN+FP}$$

## 4. Proposed Methodology

The proposed methodology, including data information, model architecture, ML models, and their assessment criteria, will be discussed in this section.

### 4.1. Novel Framework

In this work, we provide a solution to tackle the problems below for the breast cancer dataset, which we found from [16,17,18,19].

- How are the data exploratory techniques (DET) be used most efficiently utilized with the prediction models for breast cancer detection?
- How can the breast cancer features help the ML models detect cancer more precisely and more scalable?

**Figure 1:**
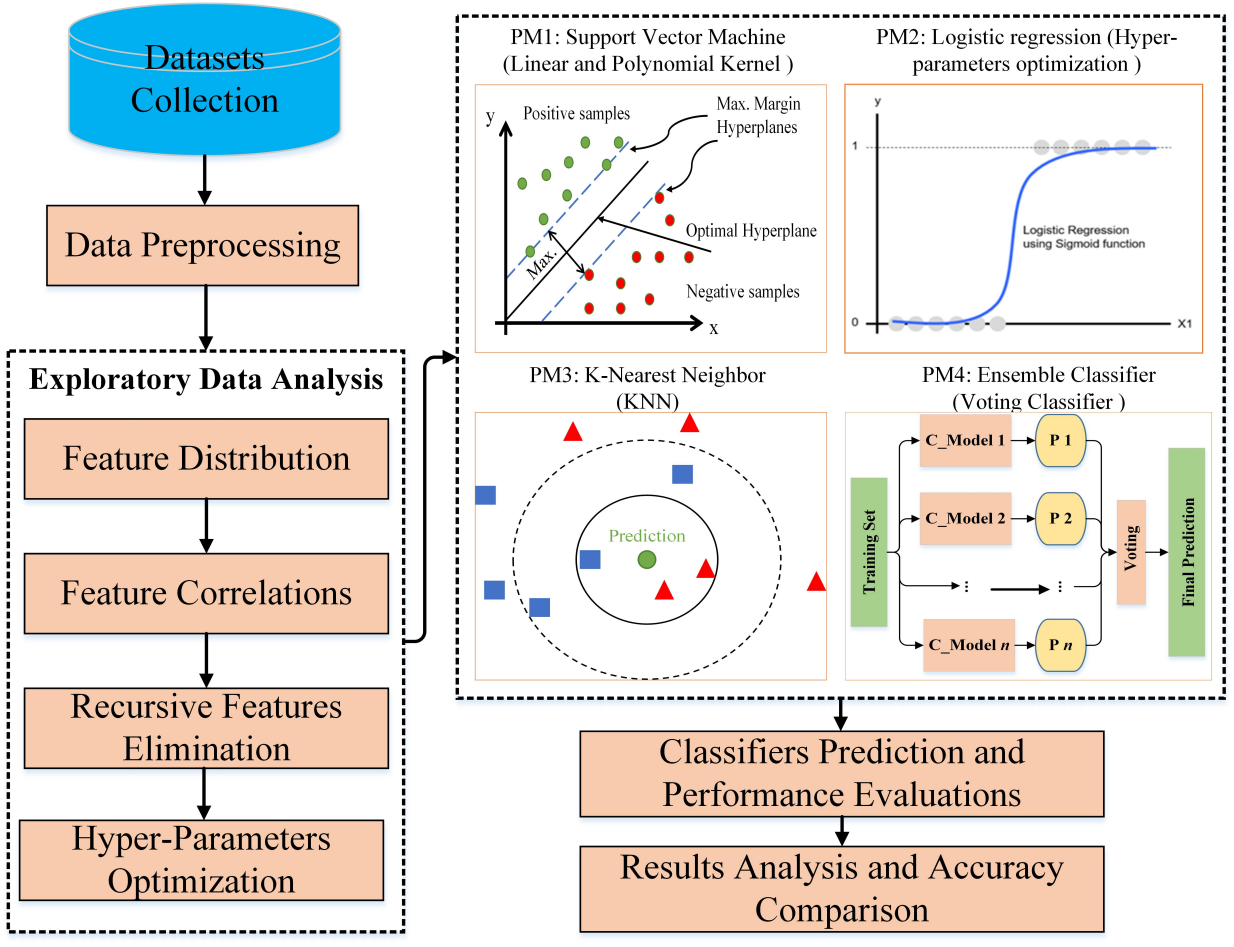
Schematic workflow diagram of our proposed method of breast cancer prediction with data exploratory techniques with machine learning classifiers.

To solve these problems, a solution is proposed, illustrated in Figure 1. This solution has nine significant different steps. The outlines of this methodology are as follows:

1. WDBC and BCCD datasets are downloaded from the machine learning repository.
2. Execute the fundamental preprocessing tasks for individual data.
3. Categorize the data into malignant and benign in WDBC and present and absent in BCCD.
4. Distribute the features into positive, negative, and random (unrelated) by calculating their correlation with each other.
5. Detect less significant features then eliminate such recursive features for effective results.
6. After exploratory data analysis, distribute the dataset into training and testing datasets.
7. Implementation of four predictive models (SVM, LR, KNN, and EC) on the datasets.
8. After the models’ execution, the classifier’s prediction is achieved with different matrices to evaluate the performance of the models, such as confusion matrices.
9. Finally, analyze the results and compare each model’s accuracy and previous research studies.

### 4.2. Data Exploratory Techniques (DET)

DE techniques, or DET, are the processes that help understand the nature of the dataset, which will identify the outliers or correlated variables that are more accessible. Our research applied feature distribution, correlation coefficient, and recursive feature elimination as our data exploratory techniques.

- **Feature Distribution:** First, the distribution of each feature was observed to find how these features are different from each other, i.e., benign and malignant in the WDBC dataset and the presence and absence of breast cancer in the BCCD dataset. The distribution was carried out by plotting the distribution plot for each feature. The data were separated by using binary code: benign (B) = 0, malignant (M) = 1, and absence = 0, presence = 1. Then, the distribution of each feature was plotted between 0 and 1.
- **Feature Correlation:** Next, the Pearson Correlation Coefficient (r) [31] calculates the correlation coefficient between each of the two features. Then, the relationship between two features can be determined by categorizing them into three groups: positively correlated features, negatively correlated features, and uncorrelated features. The features will positively correlate (*r* = +1) if the variables move in the same direction. In contrast, if these features move in the opposite direction, they will negatively correlate (*r* = −1).
- **Recursive features elimination (RFE):** RFE is one of the essential processes of machine learning. Since the dataset has many features, selecting the number of features that give the most optimal prediction result is important for improving model performance. Using fewer features that provide better understanding is the gist of doing RFE. It will recur the loop until it can find the optimal number of features. In this study, RFE was utilized to reduce the features from 30 to 15. It was conducted by a built-in function, *selector.fit(x,y)*, of *sklearn*. The attributes *support_*, and *ranking_* were passed to the ranking position of *i* − *th* feature and mask the selected features. RFE works by searching for a subset of features by starting with all features in the training dataset and successfully removing features until the desired number remains. This is achieved by fitting the given machine learning algorithm used in the core of the model, ranking features according to their relevance, discarding the least important features, and re-fitting the model. This process is repeated until a predetermined number of features is retained.
- **Hyperparameter Optimization:** Hyperparameters optimization is a process of machine learning used for tuning a set of optimal parameters. The values of these parameters are used to control the learning process. There are many approaches for hyperparameters optimization, such as grid search, random search, Bayesian optimization, gradient-based optimization, and evolutionary and population-based optimization. In this study, we used grid search optimization due to its effective results for optimization. It applies the brute-force method to generate candidates from the grid of parameter values specified with the parameter. The grid search goal is to get the highest cross-validation metric scores. In our case, we utilized *scikit* − *learn* based GridSearch K-fold CV due to the disease prediction datasets. In all prediction models, GridSearch CV was adopted to evaluate the hyperparameters. GridSearchCV uses a different combination of specified hyperparameters and their values to perform the analysis. We utilized *estimator*, *param_grid*, *scoring*, *verbose*, and *n_j_obs* parameters to calculate each combination’s performance.

### 4.3. Predictive Models

In this study, four ML classifiers were utilized as predictive models (PM) to diagnose Y-variable in the data as malignant or benign in the WDBC dataset and as the presence or absence of breast cancer in the BCCD dataset. The data were distributed into training and test sets. In experiments, we conducted this distribution by setting an integer value for the *random_state*. To tune the hyperparameter, this value can be any value, but *split_size* should be a particular value. In our scenario, we considered 20% testing sets and 80% training sets. The models were constructed on the training dataset, and then a test dataset was used to evaluate the model’s performance. We chose SVM due to its highest accuracy in the previous literature, and LR had the best performance by tuning the hyperparameter. Likewise, KNN was selected due to effective results with input features. Meanwhile, we experimented with the ensemble-based classifier using voting techniques to assess its performance and compared it with other classifiers. The precise details of these models are given below:

**PM1—SVM:** The first model applied SVM as a predictive model. SVM is one of the robust supervised machine learning algorithms used to solve classification and regression tasks [32]. The idea of SVM is to find an optimal hyperplane that gives the maximum margin of each data class (0 and 1 in this case). The SVM approach aims to solve this quadratic problem by finding a hyperplane in the high dimensional space and the classifier in the original space, as shown in (5) [33].

(5) $$\min_{\alpha }Q_{1}\left(\alpha \right)=\sum_{i=1}^{N}\alpha_{i}-\frac{1}{2}\sum_{i,j=1}^{N}\alpha_{i}\alpha_{j}y_{i}y_{j}K\left(x_{i},x_{j}\right)$$

where $K\left(x_{i},x_{j}\right)=\left(\phi \left(x_{i}\right),\phi \left(x_{j}\right)\right)$ is called the kernel function.

In this work, SVM is applied to predict whether the data are located in class 0 or 1 based on several features and then calculates its performance. SVM has many kernel functions. For the linear dataset, it is called linear kernel SVM. For nonlinear, there are many types, such as polynomial kernel SVM, radial kernel SVM, and hyperbolic tangent SVM. In this research, two different kinds of SVM kernels, the linear and polynomial kernel, were applied.

**PM2—LR:** Our second model applied LR to predict the outcomes. LR is one of the most widespread machine learning techniques. It is mainly used to predict a binary variable with a large number of independent variables. It is efficient to forecast the probability of being 0 or 1 based on predictors [34,35,36]. It can be expressed as (6):

(6) y=π(X)+ε

where *X* is a vector that contains $x_{i},i=1,2,\ldots ,n$ independent predictor variables; π(X) is the conditional probability of experiencing the event Y=1 given the independent variable vector *X*; and ε is a random error term. We can express π(X) as (7):

(7) $$\pi \left(X\right)=P\left(Y=1|X\right)=\frac{e^{X^{T}}\beta }{1+e^{X^{T}}\beta }$$

where β is the model’s parameters vector.

This study applied LR to predict whether the data are located in class 0 or 1 and then calculated the performance. LR is like an upgraded version of linear regression. However, by using linear regression to predict binary classification, some predictions will have values more than one or less than 0. A sigmoid function is employed in LR to normalize the prediction to be between 0 and 1.

**PM3—KNN:** The third model applied KNN as a predictive model. The KNN algorithm used in our problem considered the output a target class. The problem was solved or classified by the majority vote of its neighbors, where the value of K was taken as a small and real-valued positive integer [37,38]. There are different methods for calculating the distance: Manhattan, Euclidean, Cosine, etc [39]. However, this study applies to Euclidean distance only. Let $\left(c_{x_{j}},c_{y_{j}}\right)$ be the centroid and $\left(x_{i},y_{i}\right)$ be the data point. The Euclidean distance can be calculated by (8):

(8) $$euclidean=\sqrt{{\left(c_{x_{j}}-x_{i}\right)}^{2}+{\left(c_{y_{j}}-y_{i}\right)}^{2}}$$

From Figure 1 (the part of PM3), there are two types of data: square and triangle; each type is referred to as a datum. The circle in the middle is the prediction. K represents a numerical value for the nearest neighbors of the output. Given K = 3, the model will find the nearest three data points to the output in the small circle. It contains two triangles and one square, so the output will be a triangle because it has more than a circle. If K = 5, the model comprises three squares and two triangles. Therefore, the prediction result of K = 5 is square. Hence, this technique will be applied to predict whether an instance is malignant or benign in the WDBC dataset and the presence or absence of breast cancer in the BCCD dataset.

**PM4—EC:** The fourth model applies the ensemble classifier method as a predictive model. It aims to maximize the precision and recall value to detect all malignant tumors in the WDBC dataset and detect all cancer presence in the BCCD dataset. Our research applied an ensemble classifier to optimize the logistic regression model [40,41]. Ensemble classifiers have many types, i.e., bagging, boosting, and voting [42]. The kind that will be used in this research is the voting classifier. A voting classifier combines various machine learning algorithms such as SVM, LR, or KNN. Then, we ran them on the same dataset to get the prediction result of each model. Finally, it will take a majority vote to make a final prediction. For example, the voting classifier trained three algorithms; algorithm 1 resulted in “1”; algorithm 2 resulted in “0”; and algorithm 3 resulted in “0”. The final result will be “0” because two of them are “0” and only one is another option.

### 4.4. Experimental Setup

This work was implemented in Jupiter Notebook with the Python language. We processed the following key steps that can assist the data analyst or physician in implementing this work for the breast cancer prediction in real-time:

1. Import the related Python libraries such as *pandas*, *NumPy*, and *sklearn* and execute the preprocessing steps to drop out the missing values.
2. Process and execute the four-layered data exploratory techniques on each dataset.
3. Definitions and calling of all related functions, such as the confusion matrix, the precision–recall curve, the ROC curve, the learning curve, and cross-validation metrics, and assess the models’ performance.
4. Implement the proposed prediction models:
  - Starting with SVM, first, it needs to define variables and the number of test and training sets (in this case is 80% and 20%, respectively). Then, define the output results and run the model using Linear and Polynomial SVM. The results would be shown in cross-validation metrics.
  - The following model is LR; after defining the variables and splitting the data, two methods were applied to find the best hyperparameter. The first one was to use GridSearchCV, and the second one was to use Recursive Feature Elimination (RFE). Then, plot the confusion matrix, ROC curve, and learning, and find cross-validation metrics were used for both methods.
  - The 3rd prediction model was KNN; we used GridSearchCV to find the best hyperparameter to run KNN and showed the confusion matrix and cross-validation metrics.
  - The final model is EC; it applied LR with EC and the voting classifier for this work. The execution steps are similar to the previous ones. The results are shown in the confusion matrix, learning curve, and cross-validation metrics.

The experimental environment and fundamental packages for implementing proposed prediction models and DE techniques are presented in Table 2.

## 5. Results Evaluations

This section will evaluate the findings of our proposed prediction models and DE techniques and compare them to prior research.

### 5.1. Exploratory Data Analysis

**Figure 2:**
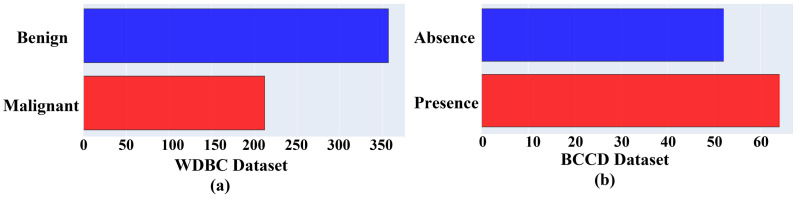
Class distributions of breast cancer datasets with the number of samples; (**a**) indicates WDBC classification into Benign and Malignant; (**b**) presents the BCCD classification into Absence and Presence.

Data exploratory techniques were discussed in the previous section, and the DE technique presents the following significant results. Figure 2 shows the size and classes of both datasets. It is obvious that the WDBC is exponentially larger than BCCD. The WDBC has benign and malignant classes (a), while the BCCD has absence and presence classes (b). Thus, proper analysis of WDBC will provide better insight. More specifically, this study focused on means, SE, worst, and correlations for demonstrating the dataset.

**Figure 3:**
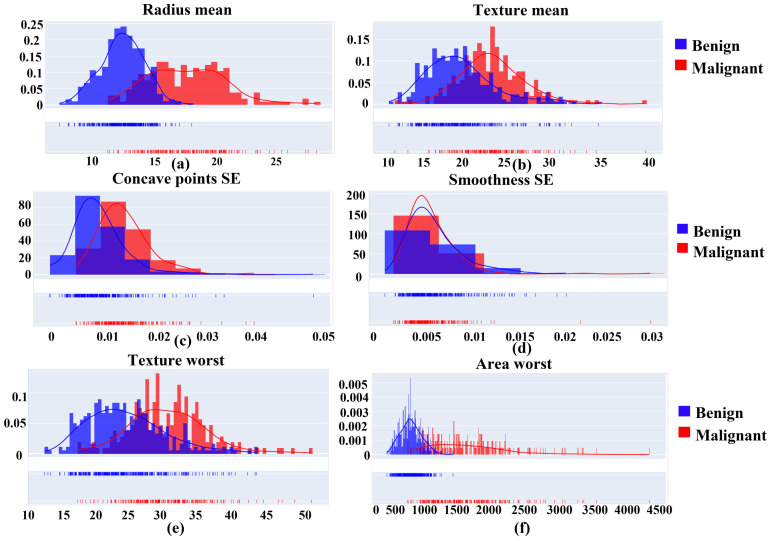
Feature distribution of WDBC dataset with samples of (**a**) Radius mean, (**b**) Texture mean, (**c**) Concave points SE, (**d**) Smoothness SE, (**e**) Texture worst, and (**f**) Area worst.

For simplicity, we presented feature distribution insights from the WDBC dataset in Figure 3. We selected two random samples from each feature. For instance, the radius means (a) from both classes of WDBC (benign and malignant) are in different shapes, and benign presented the maximum intensity. While in texture mean (b), the intensity level in both classes was almost identical in shape. Likewise, it explains the SE analysis of feature sets based on concave points and smoothness. The graphs (c) of concave up and down for both benign and malignant were different, while the inflection point was crossing the up and down moments. However, in smoothness SE (d), concave down and up were approximately in the same ranges, while malignant slopes were higher than benign. It presents the worst feature (e) and (f) distribution based on Texture and area. Here Texture waves for both benign and malignant look alike in appearance. Again, in the case of the area graph, malignant cells are flatter and more prolonged.

Furthermore, we delivered the rest of the feature’s curves in Note 01 in the Supplementary Materials.

**Figure 4:**
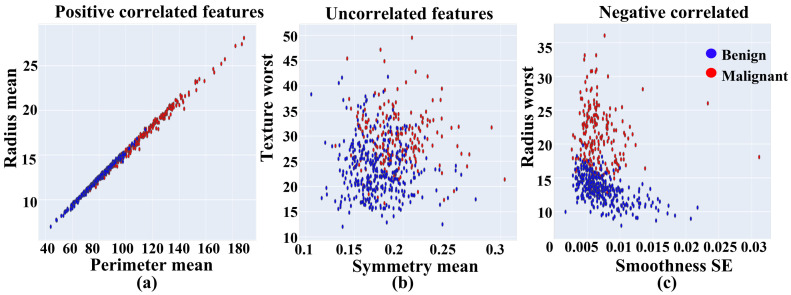
Feature correlation among different samples into positive, negative, and un-correlation of (**a**) Perimeter mean, (**b**) Symmetry mean, and (**c**) Smoothness SE respectively.

Furthermore, Figure 4 shows the feature correlations based on positively correlated features (proportional relationship) (a), uncorrelated features (no relationship) (b), and negatively correlated features (inversely proportional relationship) (c) among different features and samples. For instance, Texture worst and Symmetry means do not have any effective correlation (b). We presented a few features matrices due to a lack of space in this study. In conclusion, the feature distribution and correlational analysis enabled the proposed prediction models to detect the tumor more precisely. It is essential to mention that 80% of the total data set was used for training, and 20% of the data set was used for testing. The correlation matrix for all features is illustrated in Note 2 (Figures S2 and S3) in the Supplementary Materials. It shows the correlation of each pair of features by using a color and value system to distinguish between positive, uncorrelated, and negatively correlated features easily. For example, according to the WDBC dataset, area mean and radius mean are positively correlated features; Texture mean and smoothness mean are uncorrelated features; and smoothness SE and radius mean are negatively correlated features. According to the BCCD dataset, insulin and HOMA are positively correlated features; leptin and MCP.1 are uncorrelated features; and resistin and adiponectin are negatively correlated features.

### 5.2. Predictive Model’s Evaluations

**Figure 5:**
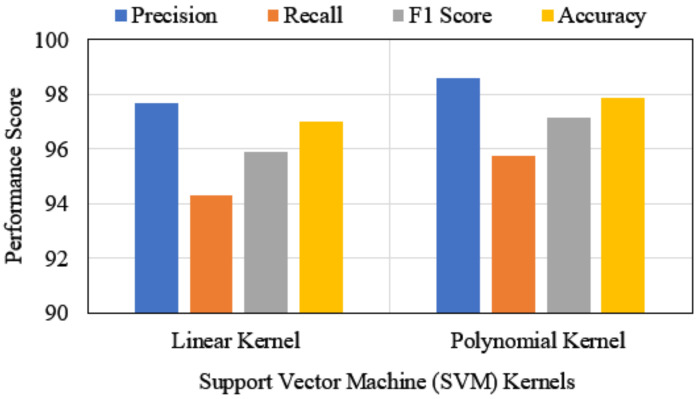
Performance’s comparison of SVM-kernels under cross-validation.

The followings are the evaluations of given predictive models (PM):

**PM1—SVM:** This work considered two kernels for employing support vector machines, i.e., linear and polynomial kernels. Table 3 shows the performance analysis of both SVM kernels with confusion matrices in which bolder entries are the highest performances. On the WDBC data set, the polynomial kernel outperformed the linear kernel on both training and testing sets. In training sets, the polynomial kernel received an almost similar precision score to the linear kernel; however, it acquired a 99.3% F1 score and a 99.12% accuracy score. On the other hand, the linear kernel performance was also significant in training and testing datasets. Similarly, the performance of the SVM model in the BCCD data set was not up to the mark. In this dataset, linear SVM succeeded with 76.91% accuracy, while the polynomial kernel had a 76.83% F1 score, which is not significant for cancer detection. For that reason, further evaluation was excluded for the BCCD dataset; and only the WDBC dataset was considered for the rest of the experiment. As the polynomial SVM kernel performance report was superior, Figure 5 is illustrated for the comparison of both kernels’ performances with four cross-validation scores with the WDBC dataset.

**Figure 6:**
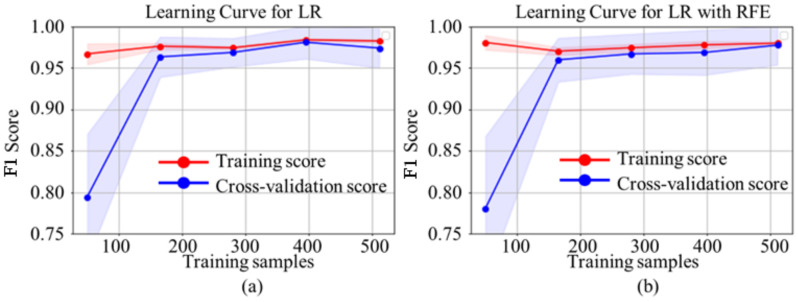
Comparisons of the learning curve of training and cross-validation scores for (**a**) simple LR and (**b**) LR with RFE.

**PM2—LR:** This study utilized three types of experiments for the LR model, i.e., basic LR, LR with 100% recall, and LR with the RFE method on the WDBC dataset. However, Figure 6 shows the comparative performance of the learning curve of LR with and without the RFE method. For the small amount of data, the training scores for both models were much more significant than the cross-validation scores. However, adding more training samples will most likely increase the generalization of the training score and cross-validation score. With the more substantial number of instances, LR with the RFE model (b) improved training and cross-validation scores. Additionally, those scores were getting closer to each other than in the simple LR model (a). Table 4 shows the cross-validation performance analysis in which bolder entries are the highest performances. Among these three methods, LR with RFE received the most significant performance, with 97.36% and 98.06% of the F1 score and accuracy values, respectively. The basic LR received the second-best performance with a slightly lower matrix score. Meanwhile, LR with 100% recall received the lowest possible scores.

**Figure 7:**
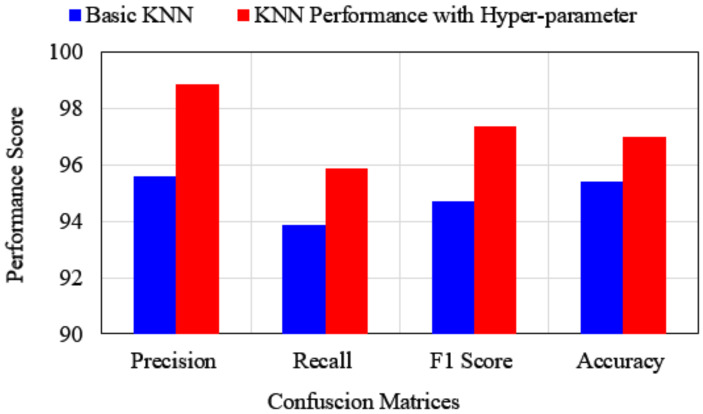
Comparison of KNN performance with basic KNN and KNN with hyperparameter.

**PM3—KNN:** The KNN predictive model has experimented on two methods, i.e., basic KNN and KNN with hyperparameter. From Figure 7, it is clear that KNN with hyperparameter showed better performance than basic KNN. Basic KNN operates automatically upon default parameters and displays results. On the other hand, hyperparameter allows parameter tuning for KNN. It represents that basic KNN acquired a 94.73% F1 score and 95.43% accuracy. Meanwhile, KNN with hyperparameter achieved a 97.35% F1 score and 97.01% accuracy.

**PM4—EC:** The performance analysis of ensemble classifiers (EC) is presented in Table 5 in which bolder entries are the highest performances. It considers three methods to evaluate the WDBC dataset: voting classifier (CV), ensemble LR, and CV prediction with 100% recall. The ensemble LR and CV achieved the highest outcomes compared to CV prediction with 100% recall. It is clear that CV successfully achieved a 96.02% F1 score while 97.61% accuracy with the given dataset. Similarly, ensemble LR performance is also significant. In contrast, CV with 100% recall values did not provide effective outcomes.

### 5.3. Classifier’s Comparative Analysis

**Figure 8:**
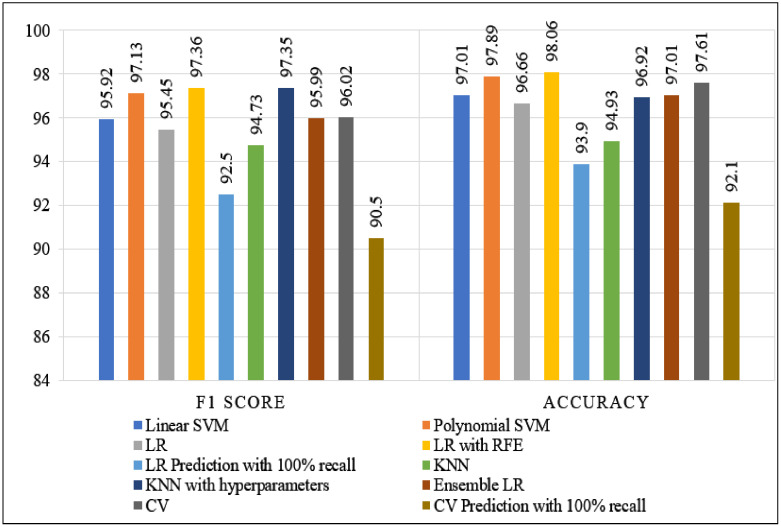
Comparison of all prediction models and methods under the cross-validation matrices.

After the individual classifier performance analysis, Figure 8 depicts the performance analysis of different classifiers and methods based on accuracy and F1 score with cross-validation matrices. The lowest performance was delivered by CV prediction with 100% recall, (basic) KNN, and LR prediction with 100% recall, where the F1 score and accuracy values were below 95%. In this comparison, LR with RFE outperformed other methods and achieved 98.06% accuracy and 97.36% F1 score. Meanwhile, polynomial SVM, CV, and KNN with hyperparameter performance are beneficial. Therefore, based on these analyses, it is clear that LR with RFE performance is higher than all other methods in cross-validation.

Furthermore, Table 6 delivers the comparison of best-achieved accuracies along with the execution time of each classifier and bolder entries are the highest performances. It presents that polynomial SVM achieves the best accuracy (99.03%) within 0.03 s, while basic KNN performed in the shortest time but with the lowest accuracy. However, LR with the RFE method performed reasonably, which had the highest accuracy (98.06%) in cross-validation. In contrast, the execution time of KNN with hyperparameter was the longest (4.023 s), although the accuracy (97.35%) was more sophisticated.

### 5.4. Comparison with Previous Studies

The best-achieved outcomes with previous studies that used the same WDBC datasets were compared. Table 7 compares the employed models or methods and the achieved accuracies in the previous studies and our proposed prediction models and outputs. The bolder entries are the outperformed results than prior works. The proposed model, SVM polynomial kernel, gained a 99.03% of accuracy, while the LR with RFE accuracy was the nearest possible 98.06% [20]. It is evident from these comparative analyses that the proposed prediction models outperformed the previous techniques and achieved sufficient accuracy for the detection of breast cancer. The possible reason for these improvements compared to other studies is the proposed data mining techniques with the ML prediction models. The DE techniques enabled the topmost accuracy while consuming the least execution time.

**Table 7:** Accuracy comparison of our proposed breast cancer prediction models with previous studies that used the same WDBC dataset.

Author Name	Reference	Year	Model/Method	Best Observed Accuracy
Maglogiannis I. et al.	[43]	2007	SVM Gaussian RBF	97.54%
Mert et al.	[15]	2015	KNN	92.56%
Hazra et al.	[29]	2016	Support Vector Machine (using 19 features)	94.423%
Osman A. H. et al.	[44]	2017	SVM	95.23%
Wang et al.	[30]	2018	SVM based ensemble learning	96.67%
Abdar et al.	[16]	2018	Nested Ensemble 2-MetaClassifier (K = 5)	97.01%
Mushtaq et al.	[18]	2019	KNN with multiple distances (Correlation K = 2)	91.00%
Rajaguru & Chakravarthy	[17]	2019	KNN Euclidean distance	95.61%
Durgalakshmi & Vijayakumar	[28]	2019	SVM	73%
Khan et al.	[45]	2020	SVM	97.06%
Al-Azzam & Shatnawi	[34]	2021	LR with area under curve	96%
Proposed Prediction Models		2022	Polynomial SVM	**99.03%**
			LR with RFE	**98.06%**
			Voting Classifier (CV)	**97.61%**
			KNN Performance with hyperparameter	**97.35%**

* The bold number indicate the top performance of the classifiers.

## 6. Discussion

Our results evaluations mostly analyzed our findings by considering the F1 score. As in real-world classification problems, large imbalanced class distributions happened in datasets. We find some observations with significant differences between the classes in the feature distribution results. For example, the concavity mean in Supplementary Note 01 had a significant difference between the distribution of benign and malignant classes. The resampling techniques, i.e., oversampling, undersampling, and cross-validation, were adopted to balance such features. The oversampling technique duplicates the minority classes, but it creates an overfitting issue for machine learning algorithms. In contrast, the undersampling technique deletes the majority classes that discard the potential data. These disadvantages can decrease machine learning accuracy for particular problems such as fraud detection, face recognition, disease detection, etc. Therefore, we omit the oversampling and undersampling techniques in our study due to the cancer detection problem. However, the author [46] suggested the cross-validation technique as a dominant technique to overcome the imbalanced class distribution. Cross-validation utilizes different portions of the data to test and train a model. This study employed the cross-validation technique using the k-fold and GridSearchCV with prediction models to balance the benign and malignant features in the training and testing dataset. The cross-validation matrices, including F1 score, precision, and recall, were compared due to the efficient use of crucial values of TP, TN, FP, and FN to deal with actual and predicted classes. The proper definitions of these metrics are given in Section 3.2.

In the polynomial SVM implementation, we secured a 99.3% F1 score, which means our proposed prediction model successfully identified the tumor and classified the cancer features as malignant. Thus, a higher F1 score means a higher diagnostic efficiency of tumors. In Table 7, this study’s F1 score and accuracy are compared with previous studies that utilized the same dataset (WDBC). These predictive models with data mining techniques would assist the data analyst in detecting the cancerous mass by analyzing the cancerous data. Similarly, Figure 8 illustrates the performance comparison of models and methods with the cross-validation techniques. As the time complexity is also a significant issue for the ML models, Table 6 presents each model’s execution time with minimum but maximum accuracy. Hence, from the above analysis, our contribution with these proposed prediction models and techniques can be efficiently helpful for the cancer domain to acquire highly satisfying results for breast cancer diagnosis.

In this study, the objective was completed for detecting breast cancer with the highest accuracy of machine learning models. However, we were unable to provide the precise reason for malignant features, which needs a domain expert. It should be noted that the BCCD dataset did not yield effective results with our prediction models except for SVM; thus, we ignored those results in this study. We provided the sources/links of the datasets in the “Data Description” subsection. As these datasets belong to American patients, the results may not be similar and effective with the Asian patients’ data. This is one of the limitations of this study, which could be extended in the future by a different dataset with neural network implementation.

## 7. Conclusions

An accurate and timely diagnosis of various diseases, i.e., breast cancer, is still a major problem for proper treatment in the healthcare field. The precise analysis of cancer features is still a time-consuming and challenging task due to the availability of massive data and the lack of DM techniques with appropriate ML classifiers. In this study, four-layered essential data exploratory techniques were proposed with four different machine learning predictive models, including SVM, LR, KNN, and ensemble classifier, to detect breast cancer tumors and classify them into benign and malignant tumors. One of the primary objectives of this study was the implementation of DE techniques before the execution of ML classifiers on the WDBC and BCCD datasets. These mining techniques enabled us to improve the prediction model’s performance with a maximum F1 score and an accuracy score higher than before. The significant finding demonstrated that the first prediction model (with an SVM polynomial kernel) had acquired the highest accuracy (99.3%). Meanwhile, logistic regression with recursive features elimination also secured 98.06% accuracy, which shows that DE techniques effectively detect higher accuracy. Our outcomes depict the competence of our prediction models for breast cancer diagnosis and provide adequate results by utilizing a short time for training the model. These sophisticated models, techniques, and results would help the physician and data analyst to apply a more intelligent classifier to diagnose breast cancer features.

As the image data relating to breast cancer are available, we will use deep learning models to detect breast cancer with novel data augmentation strategies and data exploratory techniques to handle the data scarcity and diversity. In the future, we will conduct experiments on the datasets from other countries and try to answer whether or not the different area patient’s data affect the model’s performance.

## Figures and Tables

**Table 1:** Features categorization of WDBC dataset.

No	Feature	No	Feature	No	Feature
1	Radius mean	11	Radius SE	21	Radius worst
2	Texture mean	12	Texture SE	22	Texture worst
3	Perimeter mean	13	Perimeter SE	23	Perimeter worst
4	Area mean	14	Area SE	24	Area worst
5	Smoothness mean	15	Smoothness SE	25	Smoothness worst
6	Compactness mean	16	Compactness SE	26	Compactness worst
7	Concavity mean	17	Concavity SE	27	Concavity worst
8	Concave pts. mean	18	Concave pts. SE	28	Concave pts. worst
9	Symmetry mean	19	Symmetry SE	29	Symmetry worst
10	Fractal dim. mean	20	Fractal dim. SE	30	Fractal dim. worst

**Table 2:** Information of our experimental environment.

No	Name	Version
1	Operating System	Windows 10 Home 64-bit (10.0, Build 19042)
2	Processor	Intel^®^ Core™ i7-9750H CPU@ 2.60 GHz
3	Python	3.6.10
4	Jupyter Notebook	6.0.3
5	Pandas	1.0.5
6	Numpy	1.17.0
7	SKlearn	0.23.1

**Table 3:** Performance Comparison of SVM kernels (Linear and Polynomial) on training and testing dataset of WDBC and BCCD.

Datasets Names	Data Distribution	SVM Kernels	Confusion Matrices			
			P	R	F1 Score	Accuracy
WDBC	Training Dataset	Linear SVM	98.95	98.22	98.57	98.68
		Polynomial SVM	98.62	100	**99.3**	**99.12**
	Testing Dataset	Linear SVM	97.14	95.77	96.45	95.61
		Polynomial SVM	97.26	100	98.61	98.25
BCCD	Training Dataset	Linear SVM	72.39	81.05	76.48	**76.91**
		Polynomial SVM	75.39	75.67	75.53	75.35
	Testing Dataset	Linear SVM	79.01	68.34	73.29	72.09
		Polynomial SVM	74.51	79.29	**76.83**	76.42

**Table 4:** Logistic regression performance with basic LR, LR predication with 100% recall, and LR with RFE under Cross-validation.

Matrices	Basic LR	LR Predication with 100% Recall	LR with RFE
Precision	97.15	86	98.58
Recall	93.87	100	96.22
F1 score	95.45	92.5	**97.36**
Accuracy	96.66	93.9	**98.06**

**Table 5:** Performance comparison of Ensemble LR, voting classifier (CV), and voting classifier prediction with 100% recall.

Matrices	CV Predication with 100% Recall	Ensemble LR	Voting Classifier
Precision	82.7	93.33	96.32
Recall	100	95.75	95.75
F1 score	90.5	95.99	**96.02**
Accuracy	92.1	97.01	**97.61**

**Table 6:** Execution time comparison of each model along with best-achieved accuracy.

Prediction Model (PM)	Classifiers with Proposed Approaches	Accuracy	Time (s)
PM1: SVM	Linear SVM	98.68	**0.03**
	Polynomial SVM	**99.03**	**0.03**
PM2: LR	Basic LR	96.66	0.266
	LR Predication with 100% Recall	93.9	0.87
	LR with RFE	**98.06**	0.483
PM3: KNN	Basic KNN	95.43	0.031
	KNN Performance with hyperparameter	**97.35**	4.023
PM4: Ensemble Classifier	Ensemble LR	97.01	0.634
	CV Prediction with 100% Recall	92.1	1.845
	Voting Classifier (CV)	**97.61**	0.611

## Data Availability

The codes are available at: https://github.com/abdul-rasool/Improved-machine-learning-based-Predictive-Models-for-Breast-Cancer-Diagnosis (accessed on 11 November 2021) and WDBC dataset at https://ftp.cs.wisc.edu/math-prog/cpo-dataset/machine-learn/cancer/WDBC/ (accessed on 11 November 2021) and BCCD dataset at https://archive.ics.uci.edu/ml/datasets/Breast+Cancer+Coimbra (accessed on 11 November 2021).

## Supplementary Materials

The following are available at https://www.mdpi.com/article/10.3390/ijerph19063211/s1, Figure S1: Feature distribution insights from the WDBC dataset into Benign and Malignant, Figure S2: The correlation matrix for all features of the WDBC dataset, Figure S3: The correlation matrix for all features of the BCCD dataset.
