# Supplementary material for: Improved Machine Learning-Based Predictive Models for Breast Cancer Diagnosis
Source: Int J Environ Res Public Health. 2022 Mar 9;19(6):3211. doi: 10.3390/ijerph19063211 (PMC8949437; doi:10.3390/ijerph19063211)
Supplement: Supplementary file 1 [file ijerph-19-03211-s001.zip › ijerph-1519923-supplementary.pdf]

Supplementary Note 01

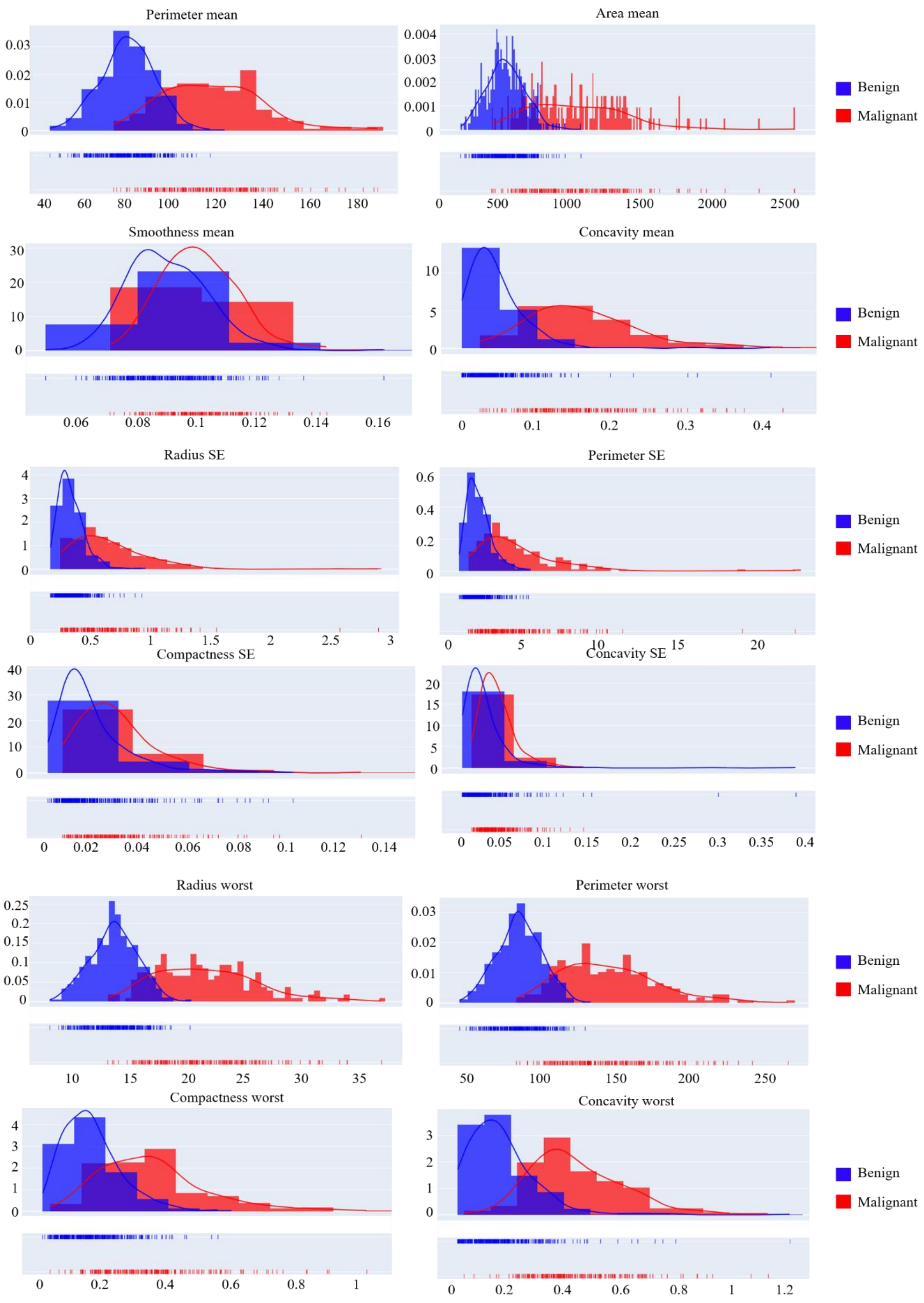

Figure S1. Feature distribution insights from the WDBC dataset into Benign and Malignant.

Supplementary Note 02

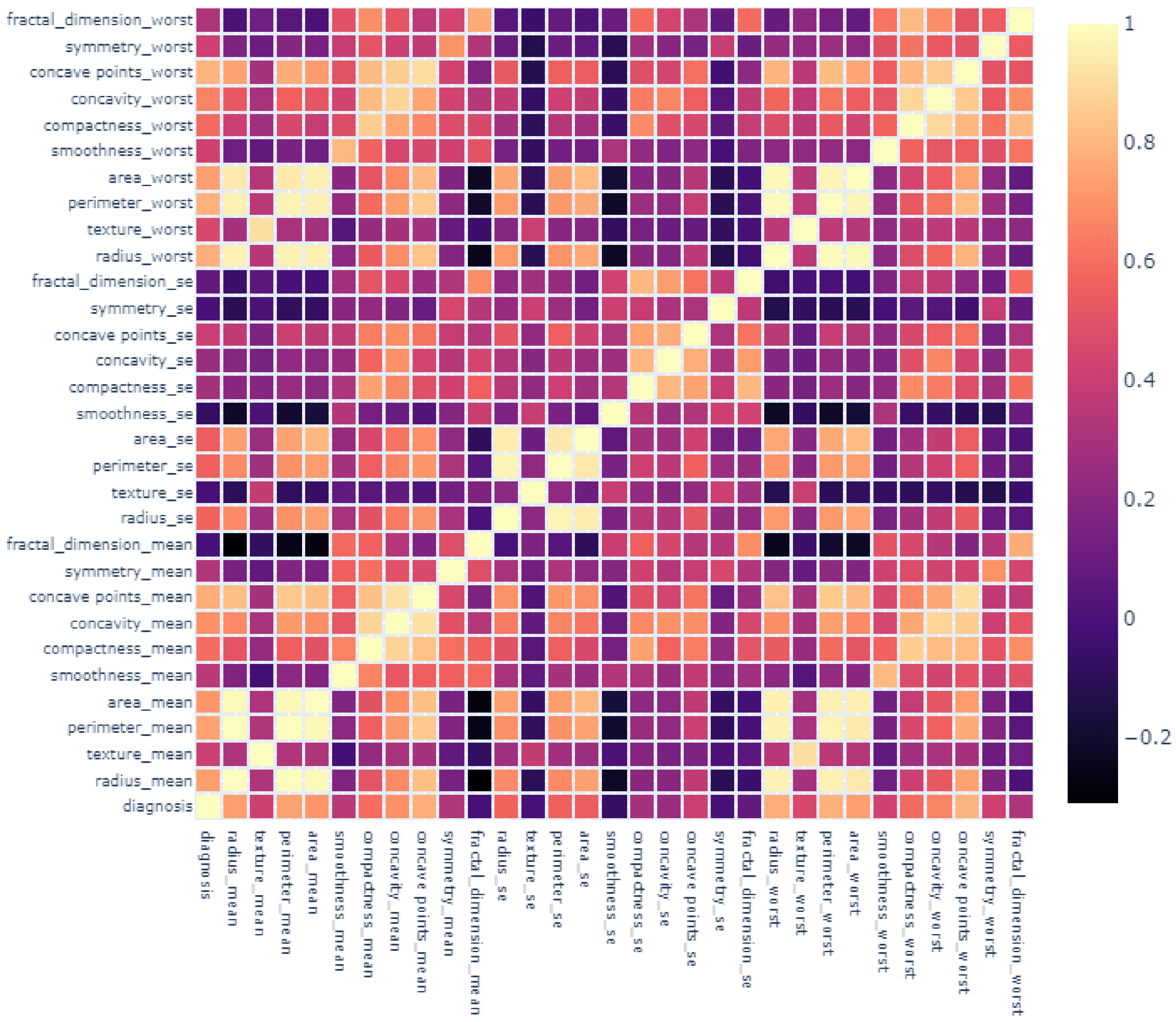

Figure S2. The correlation matrix for all features of the WDBC dataset.

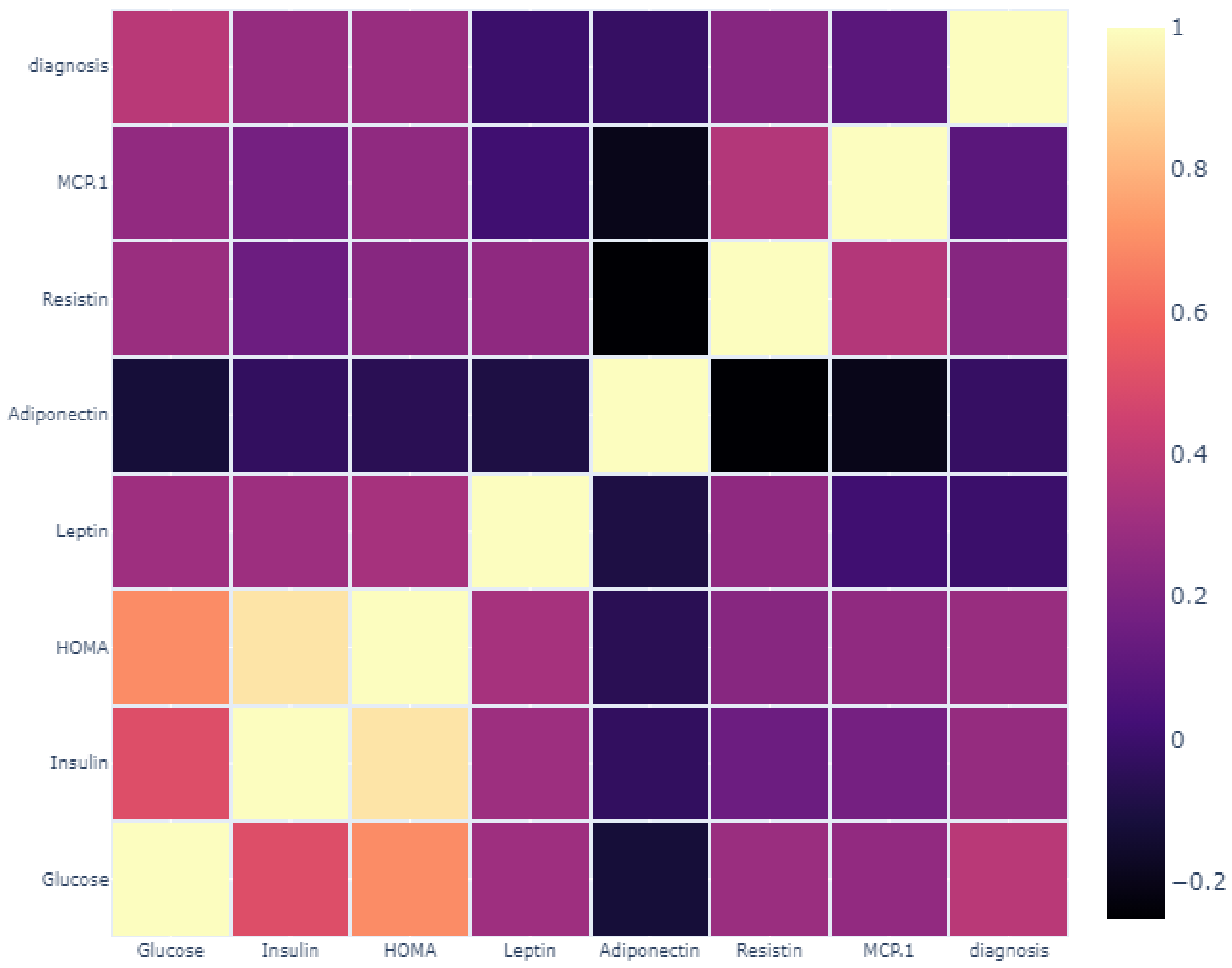

Figure S3. The correlation matrix for all features of the BCCD dataset.
